# Supplementary figures and images for: Regulation of Oncogenic Targets by the Tumor-Suppressive miR-139 Duplex (miR-139-5p and miR-139-3p) in Renal Cell Carcinoma
Source: Biomedicines. 2020 Dec 12;8(12):599. doi: 10.3390/biomedicines8120599 (PMC7764717; doi:10.3390/biomedicines8120599)

Supplemental Figure 1

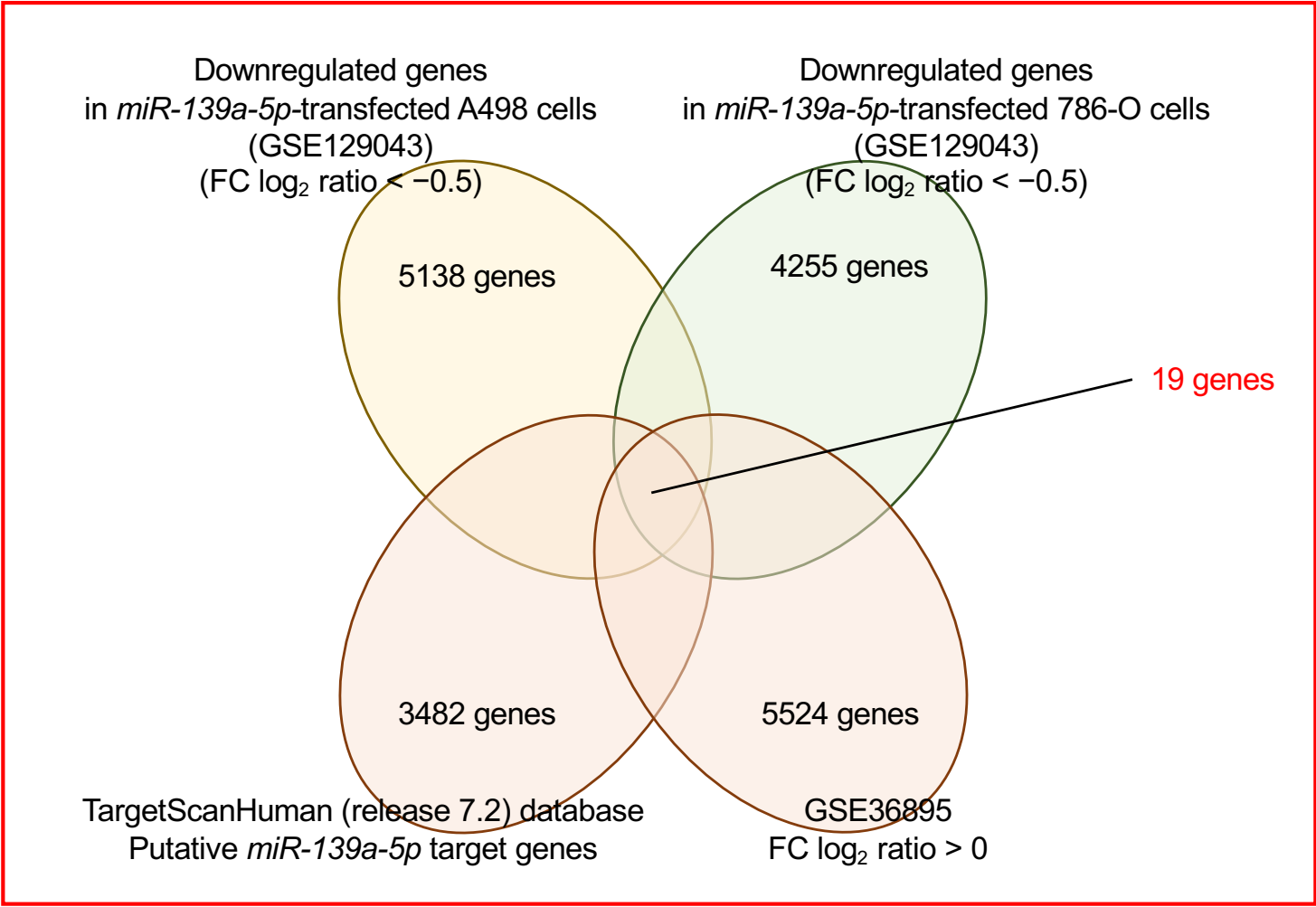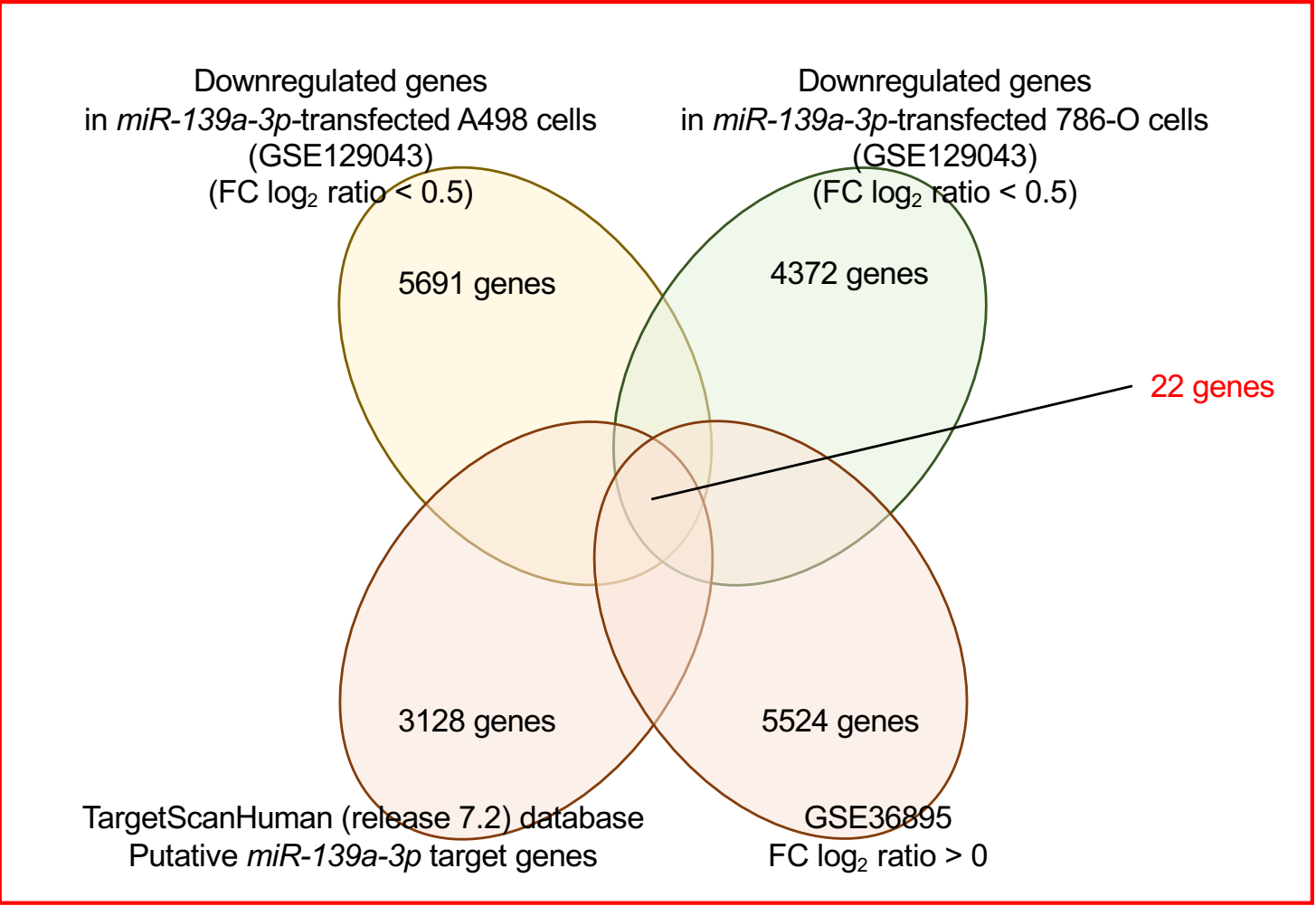

Supplemental Figure 2

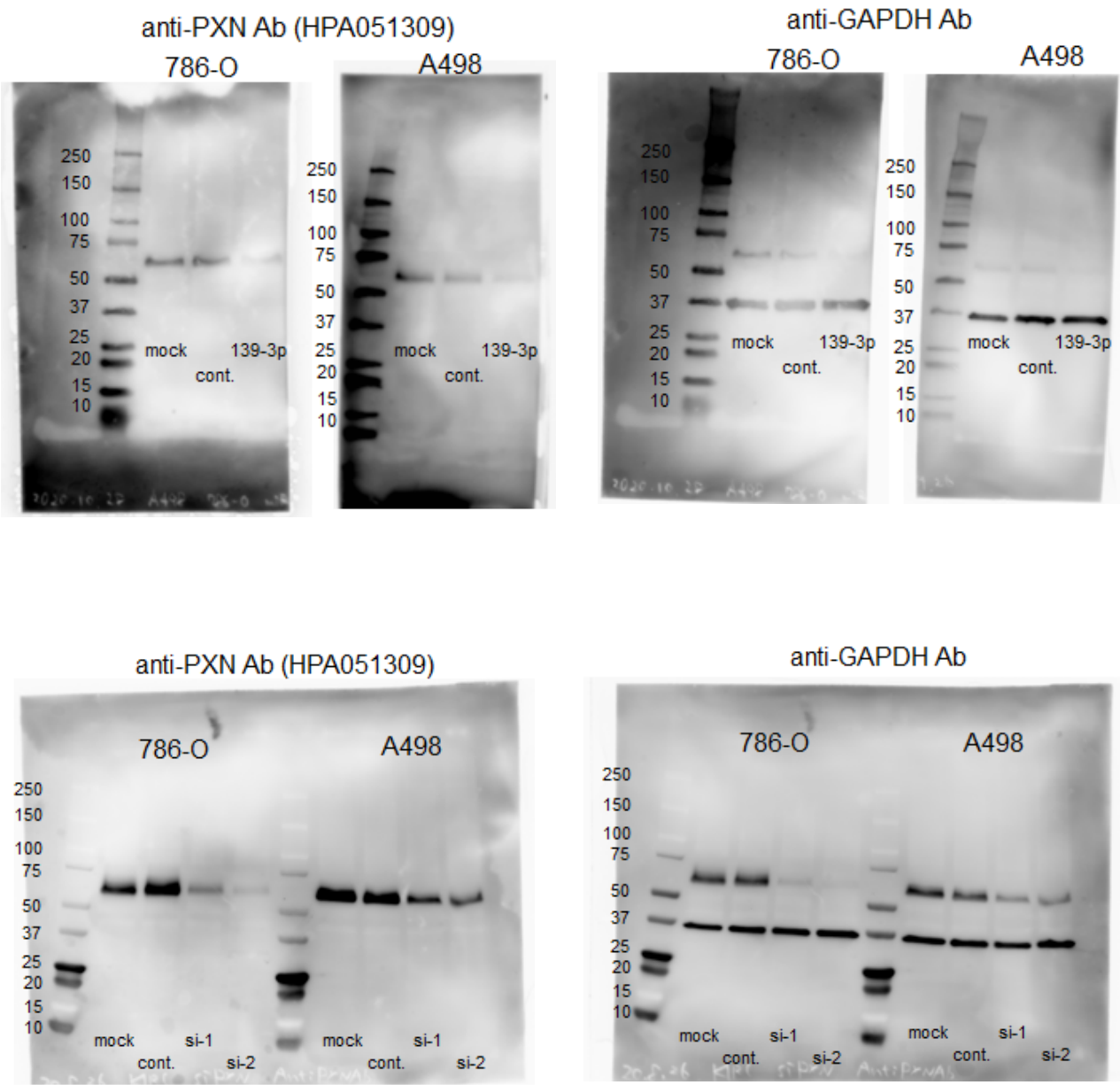

Supplement: Supplementary file 1 [file biomedicines-08-00599-s001.zip › supplementary/suppl fig.pdf]
